# Genetic associations between post-traumatic stress disorder and REM-sleep behavior disorder

**DOI:** 10.1038/s41531-026-01397-6

**Published:** 2026-05-21

**Authors:** Morvarid Ghamgosar Shahkhali, Lang Liu, Mohammad H. Ghamgosar Shahkhali, Eric Yu, Farnaz Asayesh, Jamil Ahmad, Meron Teferra, Isabelle Arnulf, Pauline Dodet, Yo-El Ju, Michele T. M. Hu, Jacques Y. Montplaisir, Jean-François Gagnon, Alex Desautels, Abubaker Ibrahim, Ambra Stefani, Birgit Högl, Merve Aktan-Süzgün, Alex Iranzo, Mónica Serradell, Angelica Montini, Gerard Maya, Carles Gaig, Gian Luigi Gigli, Mariarosaria Valente, Francesco Janes, Andrea Bernardini, Yves Dauvilliers, Karel Sonka, David Kemlink, Petr Dusek, Michael Sommerauer, Gültekin Tamgüney, Michela Figorilli, Monica Puligheddu, Valerie Cochen De Cock, Wolfgang Oertel, Annette Janzen, Elena Antelmi, Brit Mollenhauer, Friederike Sixel-Doring, Michele Terzaghi, Giuseppe Fiamingo, Matej Skorvanek, Kristina Kulcsarova, Beatriz Abril, Luigi Ferini-Strambi, Jitka Bušková, Beatrice Orso, Pietro Mattioli, Dario Arnaldi, Femke Dijkstra, Mineke Viaene, Bradley F. Boeve, Owen A. Ross, Guy A. Rouleau, Lee E. Neilson, Jonathan E. Elliott, Miranda M. Lim, Ronald B. Postuma, Ziv Gan-Or

**Affiliations:** 1 The Neuro (Montreal Neurological Institute-Hospital), McGill University, Montréal, QC Canada; 2 Department of Human Genetics, McGill University, Montréal, QC Canada; 3 Department of Neurology and Neurosurgery, McGill University, Montréal, QC Canada; 4 Department of Computer Engineering, Ahrar Institute of Technology and Higher Education, Rasht, Iran; 5 Sleep Clinic, Pitié Salpêtrière Hospital, APHP-Sorbonne University and Paris Brain institute, Paris, France; 6 Department of Neurology, Washington University School of Medicine, St. Louis, MO USA; 7 Nuffield Department of Clinical Neurosciences, University of Oxford, Oxford, UK; 8 Centre d’Études Avancées en Médecine du Sommeil, Hôpital du Sacré-Coeur de Montréal, Montréal, QC Canada; 9 Department of Psychiatry, Université de Montréal, Montréal, QC Canada; 10 Department of Psychology, Université du Québec à Montréal, Montréal, QC Canada; 11 Department of Neurosciences, Université de Montréal, Montréal, QC Canada; 12 Department of Neurology, Sleep Disorders Clinic, Medical University of Innsbruck, Innsbruck, Austria; 13 Neurology Service, Sleep Centre, Hospital Clínic Barcelona, Universitat de Barcelona, IDIBAPS, CIBERNED: CB06/05/0018-ISCIII, Barcelona, Spain; 14 Department of Head, Neck and Neurosciences, Clinical Neurology Unit, University Hospital of Udine, Udine, Italy; 15 Department of Medicine (DMED), University of Udine, Udine, Italy; 16 National Reference Centre for Orphan Diseases, Narcolepsy—Rare Hypersomnias, Sleep Unit, Department of Neurology, CHU Montpellier, Institute for Neurosciences of Montpellier INM, Univ Montpellier, INSERM, Montpellier, France; 17 Department of Neurology and Centre of Clinical Neuroscience, Charles University, First Faculty of Medicine and General University Hospital, Prague, Czech Republic; 18 Department of Neurology, Faculty of Medicine and University Hospital Cologne, University of Cologne, Cologne, Germany; 19 Cognitive Neuroscience, Institute of Neuroscience and Medicine (INM-3), Research Centre Jülich, Jülich, Germany; 20 Center for Neurology, Department of Parkinson, Sleep, and Movement Disorders, University Hospital of Bonn, Bonn, Germany; 21 Institute of Biological Information Processing—Structural Biochemistry (IBI-7), Forschungszentrum Jülich, Jülich, Germany; 22 Heinrich-Heine-Universität Düsseldorf, Mathematisch-Naturwissenschaftliche Fakultät, Institut für Physikalische Biologie, Düsseldorf, Germany; 23 Department of Medical Sciences and Public Health, Sleep Disorder Research Center, University of Cagliari, Cagliari, Italy; 24 Sleep and Neurology Unit, Beau Soleil Clinic, Montpellier, France; 25 EuroMov Digital Health in Motion, Univ Montpellier, IMT Mines Ales, Montpellier, France; 26 Department of Neurology, Philipps-University, Marburg, Germany; 27 AOVR, Verona, Italy; 28 DIMI Department of Engineering and Medicine of Innovation, University of Verona, Verona, Italy; 29 Department of Neurology, University Medical Centre Göttingen, Göttingen, Germany; 30 Paracelsus-Elena-Klinik, Kassel, Germany; 31 Department of Brain and Behavioral Sciences, University of Pavia, Pavia, Italy; 32 Department of Neurology, Faculty of Medicine, P. J. Safarik University, Kosice, Slovakia; 33 Department of Neurology, University Hospital of L. Pasteur, Kosice, Slovakia; 34 Department of Clinical Neurosciences, Faculty of Medicine, P. J. Safarik University, Kosice, Slovakia; 35 Sleep Disorder Unit, Carémeau Hospital, University Hospital of Nîmes, Nîmes, France; 36 Department of Neurological Sciences, Università Vita-Salute San Raffaele, Milan, Italy; 37 Department of Sleep Medicine, National Institute of Mental Health, Klecany, Czech Republic; 38 Third Faculty of Medicine, Charles University, Prague, Czech Republic; 39 Department of Neuroscience (DINOGMI), University of Genoa, Genoa, Italy; 40 IRCCS Ospedale Policlinico San Martino, Genoa, Italy; 41 Laboratory for Sleep Disorders, St. Dimpna Regional Hospital, Geel, Belgium; 42 Department of Neurology, St. Dimpna Regional Hospital, Geel, Belgium; 43 Department of Neurology, Antwerp University Hospital, Edegem, Belgium; 44 Department of Neurology, Mayo Clinic, Rochester, MN USA; 45 Departments of Neuroscience and Clinical Genomics, Mayo Clinic, Jacksonville, FL USA; 46 Department of Neurology, Oregon Health & Science University, Portland, OR USA; 47 Department of Veteran Affairs, VA Portland Health Care System and VISN20 Northwest Mental Illness Research, Education, and Clinical Center, Portland, OR USA

**Keywords:** Diseases, Genetics, Neurology, Neuroscience

## Abstract

Isolated/idiopathic rapid-eye movement (REM) sleep behavior disorder (iRBD) is, in most cases, an early form of α -synuclein-related neurodegenerative diseases, including Parkinson’s disease and dementia with Lewy bodies. Clinical reports suggest that iRBD is more common in individuals with Post-Traumatic Stress Disorder (PTSD) compared to those without PTSD. We conducted polygenic risk score (PRS), genetic correlation, and Mendelian randomization analyses to explore potential genetic and/or causal associations between PTSD and iRBD. Dopamine transporter imaging binding status was also examined in iRBD patients with (*N* = 6) and without PTSD (*N* = 32). While not supporting a causal relationship, genetic analyses revealed a significant association between PTSD and iRBD, consistent with the exploratory imaging substudy. These findings suggest that individuals genetically at risk for PTSD may also be at higher risk for iRBD. Further investigation of iRBD in individuals with PTSD may help inform potential neurodegenerative risk.

## Introduction

Isolated/idiopathic rapid eye movement (REM) sleep behavior disorder (iRBD) is a parasomnia characterized by the lack of normal muscle paralysis during REM sleep, leading individuals to act out their dreams. Symptoms can vary from non-violent expressions like laughing and crying to more aggressive actions such as kicking or punching, which can potentially cause harm to both the individual and their sleep partner. iRBD is also an early clinical indicator of α-synucleinopathies and over 80% of individuals with iRBD will eventually develop either Parkinson’s disease (PD), dementia with Lewy bodies, and more rarely, multiple system atrophy. This association highlights the importance of investigating iRBD in the context of neurodegeneration^1^.

Previous clinical reports suggest a potential association between iRBD and post-traumatic stress disorder (PTSD). A report showed an early association between PTSD and RBD, where 56% (*n* = 14/27) of individuals with RBD had a history of PTSD^2^. This was later reinforced through a cohort study of 394 United States Veterans, reporting an age-adjusted odds ratio of 3.4 associating the presence of PTSD with RBD, corresponding to an overall frequency rate of 15–20%^3^. Similarly, a study found that compared to individuals who suffered from trauma but did not develop PTSD, individuals with PTSD had ~10–15 fold risk for RBD^4^. In parallel, several prospective longitudinal or retrospective pseudolongitudinal cohort studies have identified links between PTSD and neurodegenerative disorders. One study found that people with PTSD had nearly twice the risk of developing dementia compared to those without PTSD^5^, while others showed an increased risk of PD later in life^6,7^. A case-control study found that people with RBD and a history of both traumatic brain injury (TBI) and PTSD showed worse synucleinopathy-related neurological measures than those with only RBD, highlighting the close relationship between these conditions and the need for further investigation of their shared mechanisms^8^.

In this study, we first aimed to examine whether there is genetic evidence for the association between PTSD and iRBD. We conducted polygenic risk score (PRS), genetic correlation and Mendelian randomization (MR) analyses to investigate potential genetic and causal relationships between PTSD and iRBD. Next, we aimed to explore dopamine transporter single-photon emission computed tomography (DaT-SPECT) binding status, a marker of neurodegeneration, in a subset of iRBD participants with and without PTSD. A blinded observer evaluated DaT-SPECT binding status as an objective marker of nigrostriatal degeneration in iRBD in consecutive participants from a single site enriched for PTSD, and comparisons between iRBD participants with and without PTSD were made, adjusting for known confounders. To our knowledge, this is the first study to investigate the genetic association between PTSD and iRBD.

## Results

### PTSD PRS is associated with increased risk of iRBD

**Fig. 1:**
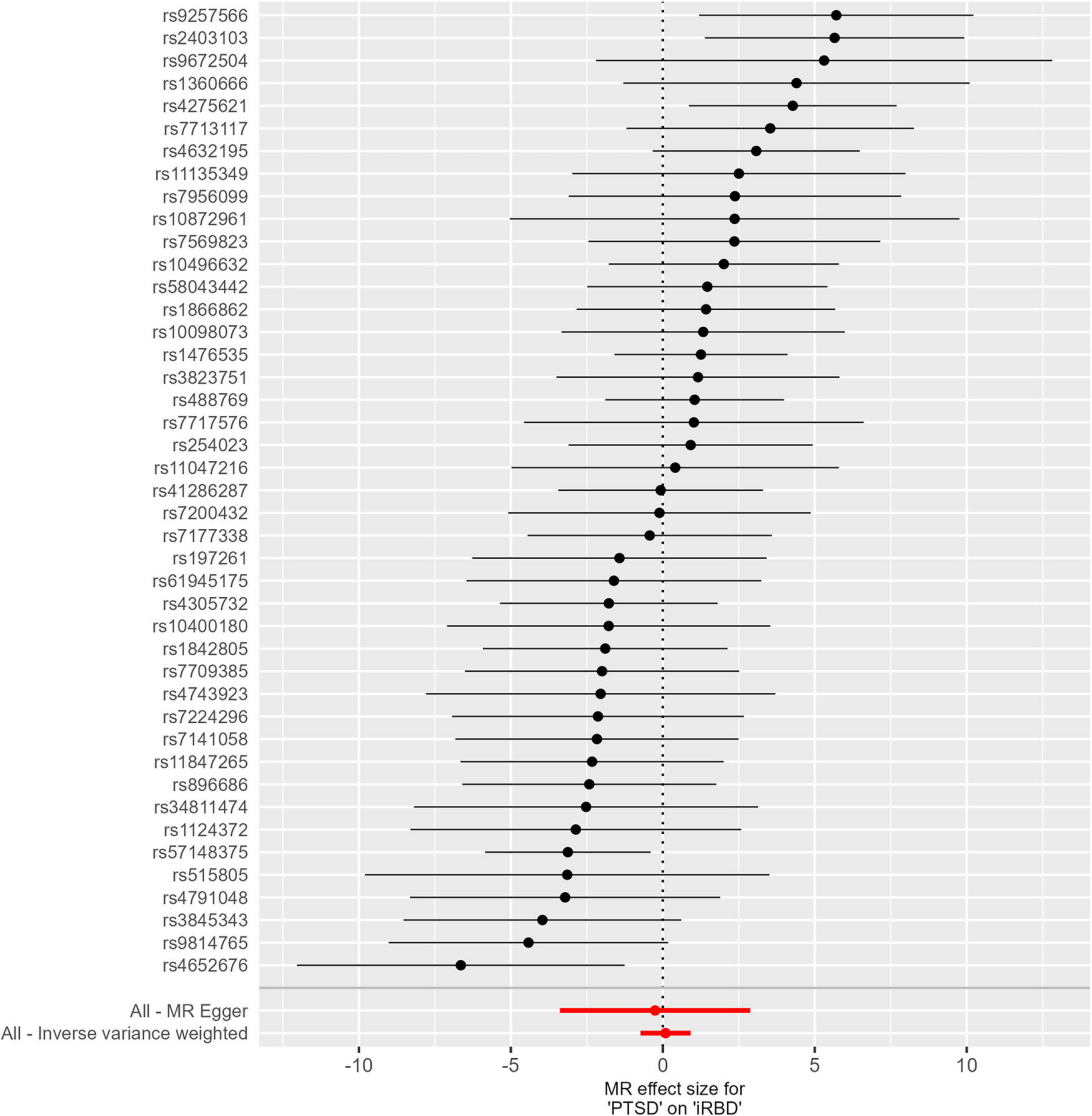
Mendelian randomization estimates for Post-Traumatic Stress Disorder (PTSD) on isolated/idiopathic rapid-eye movement (REM) sleep behavior disorder (iRBD). The figure showes the causal effect estimates for individual genetic variants and the combined effects obtained using inverse variance weighted (IVW) and MR Egger methods. Error bars represent 95% confidence intervals.

We examined the potential genetic association between PTSD PRS and iRBD using a logistic regression model. Individuals with a higher genetic risk for PTSD were more likely to develop iRBD, as for each one standard deviation (SD) increase in the standardized PTSD PRS, the odds of having iRBD increased by 14.7% (odds ratio (OR) = 1.15, 95% confidence interval (CI): 1.04 to 1.26, *p* = 0.005, Nagelkerke’s *R*² = 0.0028). This suggests that there is a significant association between genetic risk for PTSD and the risk of iRBD. We then reran the model including lifetime major depressive disorder (MDD) PRS as an additional covariate. PTSD PRS remained significantly associated with iRBD (OR = 1.14, 95% CI: 1.04–1.26, *p* = 0.007, Nagelkerke’s *R*² = 0.0026), indicating that the association was not fully explained by shared genetic liability with depression. However, using linkage disequilibrium score regression (LDSC), we found a weak positive genetic correlation of only 0.03 (standard error (SE) = 0.02) that was not statistically significant (*p* = 0.11, Table 1). We performed MR analysis to investigate a potential causal relationship between PTSD and iRBD. Using an inverse variance weighted approach with 43 PTSD genetic instruments, we obtained a beta estimate of 0.09 (SE = 0.42, *p* = 0.830) in the PTSD-to-iRBD direction (Table 2 and Fig. 1). Furthermore, in the iRBD-to-PTSD direction, only two significant single-nucleotide polymorphisms (SNP) remained, limiting statistical power and precluding robust conclusions in that direction.

**Table 1:** Summary of analytical methods and results

Analysis	Tool/Method	Estimate	Standard error	*p* value
PRS Analysis	PRS-CS	*β*: 0.137	0.049	0.005
Genetic Correlation	LDSC	rg: 0.031	0.019	0.106
Mendelian Randomization	TwoSampleMR, IVW	*β*: 0.0907	0.422	0.830

*PRS* polygenic risk score, *PRS-CS* polygenic risk score with continuous shrinkage, *LDSC* linkage disequilibrium score regression, a statistical method used to estimate genetic correlation while accounting for linkage disequilibrium, *IVW* inverse variance weighting, a Mendelian randomization method that combines effect estimates from multiple genetic variants, *β* beta coefficient, the estimated effect size in the statistical model, *rg* genetic correlation, a measure of the shared genetic basis between two traits.

**Table 2:** Mendelian randomization analysis

Method	*N* SNP	*β*	SE	*p* value
MR Egger	43	−0.2541169	1.5987146	0.8744881
Weighted median	43	0.340319	0.5242078	0.516205
Inverse variance weighted	43	0.0907476	0.422071	0.829763
Simple mode	43	−2.0768048	1.3482727	0.1309776
Weighted mode	43	1.1385041	1.1450945	0.3257976

*MR* Mendelian randomization, *N SNP* number of single nucleotide polymorphisms used in the analysis, *β* beta coefficient, the estimated effect size in the statistical model, *SE* standard error.

### Individuals with iRBD and PTSD have higher rates of abnormal DaT-SPECT

Further exploring the association between PTSD, RBD, and potential increased risk of neurodegeneration, we performed a sub-analysis of neuroimaging data from iRBD participants co-enrolled in the North American Prodromal Synucleinopathy (NAPS) Consortium^9^, from a single site enriched for PTSD. Of 38 participants with RBD who underwent DaT-SPECT, there was a significant increase (*p* = 0.01, *X*^2^ = 6.62) in the ratio of an abnormal DaT-SPECT status in those with PTSD (*n* = 5/6 with PTSD showed abnormal DaT-SPECT; 83%) compared to those without PTSD (*n* = 9/32 without PTSD showed abnormal DaT-SPECT; 28%). This significant association for PTSD (OR = 189.2, 95% CI: 8.2–34,223) was maintained after correcting for age (OR = 1.26, 95% CI: 1.08–1.61), sex (OR = 0.75, 95% CI: 0.02–14.5), history of traumatic brain injury (OR = 6.34, 95% CI: 0.66–135.6), and years since RBD symptom onset (OR = 0.91, 95% CI: 0.79–0.98) via multiple logistic regression. The average time since RBD symptom onset in those with and without PTSD was 35.8 ± 15.7 vs. 21.8 ± 18.5 years, respectively (*p* = 0.09; 95% CI: −30.8 to 2.6; unpaired two-tailed heteroscedastic *t*-test).

## Discussion

PRS analysis demonstrated a positive genetic association between PTSD PRS and the risk of developing iRBD. In other words, individuals with higher genetic risk for PTSD also have higher risk for iRBD consistent with the neuroimaging sub-analysis. However, genetic correlation analysis did not indicate a strong shared genetic architecture, and no evidence of a potential causal relationship was observed using MR. The absence of a genetic correlation and causal link between PTSD and iRBD may be due to the limited power stemming from the small sample size of the iRBD genome-wide association studies (GWAS). Identifying most genetic variants in highly polygenic traits requires large sample sizes to achieve genome-wide significance and capture the majority of genetic variance. Additionally, genetic correlation and MR depend on stable effect estimates and strong instruments, which face challenges in identifying strong and valid instruments for highly polygenic traits. In contrast, PRS analysis is particularly powerful for detecting associations between polygenic traits by aggregating numerous SNP effects, even when individual effect sizes are small.

The relationship between PTSD, iRBD, and neurodegeneration is supported by several key studies. In one study of 394 veterans, researchers used in-lab polysomnography (PSG) and self-reported dream enactment to find that the overall prevalence of iRBD was 15% among those with PTSD^3^. In comparison, the prevalence of iRBD in the general population is estimated to be approximately 1%^1^. Also, Veterans with PTSD were approximately 2.8 times more likely to develop RBD, with a 147% increase in the prevalence ratio after age adjustment^3^. A retrospective study found that patients with PTSD had significantly higher REM sleep without atonia (RSWA) across multiple metrics compared to controls (all *p* < 0.025)^10^.

One plausible biological system underlying the observed genetic overlap between PTSD and iRBD is dysregulation of arousal and REM sleep control. Polysomnographic studies in PTSD consistently report alterations in REM sleep, including changes in REM timing, intensity, and proportion, along with reduced arousals from non-REM sleep. In addition, patients with PTSD show increased phasic and tonic muscle activity during REM sleep, closely resembling the electrophysiologic features of RBD. Neuropathologic evidence further suggests reduced locus coeruleus function in PTSD, leading to downstream brainstem dysregulation, a pathophysiologic mechanism that is also implicated in RBD and may contribute to shared vulnerability across the two conditions^2^.

Beyond iRBD, PTSD is also a recognized independent risk factor for neurodegenerative diseases, particularly PD and Alzheimer’s disease (AD). A large cohort study of 181,093 midlife veterans showed that those with PTSD were 2.31 times more likely to develop dementia, including all dementia subtypes such as AD and Lewy body dementia^5^. A longitudinal study found individuals with PTSD had a higher likelihood of developing PD (2% vs. 0.5%) and a shorter interval between PTSD diagnosis and PD development^6^. Another study reported that PTSD was associated with a 1.5- to 1.9-fold increased risk of PD over successive 5-year periods^7^. A study of 24 individuals with both TBI and PTSD (neurotrauma) and RBD showed significantly greater impairments in cognitive, motor, and autonomic function compared to 96 individuals with only RBD, suggesting the combination may be linked to a more advanced neurodegenerative process or altered neuropathogenic trajectory^8^. Importantly, known PD risk genes such as *GBA1* and *LRRK2* do not appear to be individually associated with PTSD^11^. This possible association between PTSD, RBD, and neurodegeneration is further explored by our observational sub-analysis of NAPS Consortium participants with DaT-SPECT. PTSD was significantly associated with an increased rate of abnormal DaT-SPECT interpretations, even after correcting for age, sex, history of brain injury, and time since RBD symptom onset. These data are consistent with clinical evidence for accelerated or altered neuropathology in iRBD with PTSD. However, we highlight the small sample sizes between groups, which should be considered in this interpretation. For example, the lack of statistical significance between PTSD+ and PTSD− participants' duration of RBD symptoms may not reflect an absence of a clinically meaningful difference. Furthermore, the employed logistic regression model is arguably over fitted using four unique predictors, a small sample size, and quasi-separation, suggesting model stability may limit the generalizability of this association. Further testing, e.g., Firth-penalized logistic regression as a sensitivity analysis retained the observed association (likelihood ratio test: *χ*² = 11.96, *p* = 0.0005). The Firth-corrected odds ratio for PTSD (126.1) was modestly attenuated relative to the standard maximum likelihood estimate (189.2), consistent with expected shrinkage under penalization, and the association remained the dominant signal in the model.

PTSD is also known to be comorbid and genetically correlated with MDD. PTSD and major depressive episode (MDE) showed a correlation of 0.50 (*p* < 0.05, two-sided), reflecting substantial comorbidity between the two disorders^12^. Genetic correlation analyses have further demonstrated a shared genetic architecture between PTSD and MDD (rg = 0.85, SE = 0.008, *p* < 2 × 10^−16^)^11^, consistent with findings from another study reporting a similarly high genetic correlation (rg = 0.80, SEM = 0.08, Pfdr = 5.06 × 10^−24^)^13^. This overlap is particularly relevant given that MDD has also been associated with RBD^14^, suggesting that part of the observed relationship between PTSD and RBD could be mediated through their shared depressive pathology. However, we found that the genetic association between PTSD and RBD is independent of MDD. Understanding whether the association between MDD and RBD reflects shared affective or regulatory biology, or an independent link with neurodegeneration, will require further investigation in future studies.

Our study has several limitations. One major limitation is the small sample size for the iRBD GWAS, which may be the reason for the limited power in detecting genetic correlations and conducting MR analyses. In addition, the sample size of the iRBD cohort used for PRS analyses was small, and this limitation could be addressed in future studies with larger cohorts. Another limitation is that our analysis focused exclusively on individuals of European ancestry, due to the limited availability of PSG-confirmed iRBD cases in other populations. This narrow scope may restrict the generalizability of our findings to non-European groups. In addition, the dataset was unbalanced, as all cases were derived from the McGill cohort, which could introduce bias related to recruitment strategies or differences in iRBD assessment across cohorts. Although the McGill cohort included multiple European subpopulations and population structure was accounted for using principal components, future studies should aim to include larger, more diverse, and better-balanced cohorts to validate these results and improve generalizability. Additionally, the lack of detailed PTSD status information in cases and controls represents a limitation, and we cannot rule out some bias as a result. RBD status was also not available for controls; however, given the low prevalence of iRBD in the general population (approximately 1%), any resulting misclassification is expected to be minimal and unlikely to materially affect the results. Future studies recording the presence of iRBD, PTSD and trauma in both patients and controls will be needed. In addition, we did not have access to the individual-level genotypes in the PTSD GWAS, therefore we could not perform an analysis examining iRBD PRS effect on PTSD risk. Moreover, although there is evidence linking TBI with both PTSD and RBD^3^, this relationship could not be investigated in the current study due to the lack of TBI-related data and PTSD status in iRBD cohort. Future studies should explore these potential associations. Because DaT-SPECT is laborious and presents high burden and cost to both participants and studies, sample sizes for these outcomes will be limited but are critically important to consider in rigorous controlling for multiple relevant covariates. Furthermore, additional work is needed that also includes other quantitative neurodegenerative outcome measures. Future studies may address these limitations and incorporate molecular and functional experiments along with longitudinal and methodological approaches to better understand the complex relationship between PTSD and RBD and to offer potential explanations for the observed association.

To conclude, our study suggests that individuals who are genetically at risk for PTSD are also at risk for iRBD, providing further support for previous clinical observations. Future exploration of iRBD identification in individuals with PTSD may help signal potential neurodegenerative risk.

## Methods

### Population

We used the most recently published GWAS summary statistics for PTSD (PGC-PTSD Freeze 3) of European ancestry (*N* cases = 137,136; *N* controls = 1,085,746)^11^, and GWAS summary statistics for iRBD (*N* cases = 1061; *N* controls = 8386)^15^ for MR and genetic correlation analyses. Additionally, we analyzed individual GWAS genotyping data from a total of 1,292 iRBD cases (80% men, average age at onset: 60 ± 13 years SD) diagnosed according to the International Classification of Sleep Disorders (2nd or 3rd Edition), including video PSG, and 67,150 controls (53% men, average age: 57 ± 9 years SD) from four major cohorts, which were used for polygenic risk score (PRS) analysis. These cohorts included the McGill cohort (*N* cases = 1292; *N* controls = 1353), comprising large cohorts of French, French Canadian, Italian, and British origins, as well as smaller cohorts from other European populations genotyped and analyzed at McGill University, NeuroGenetics Research Consortium (NGRC; dbGap phs000196.v3.p1; *N* controls = 1968)^16^, National Institute of Neurological Disorders and Stroke (NINDS) Genome-Wide genotyping in Parkinson’s Disease (dbGap phs000089.v4.p2; *N* controls = 790)^17^, and UK Biobank (UKB; *N* controls = 63,039, Table 3)^18^. Population substructure was accounted for by including principal components as covariates in subsequent analyses. Data on PTSD were not available for the iRBD cohort included in this study. Moreover, we explored with DaT-SPECT a sub-analysis of participants with iRBD (*N* = 6 with PTSD; *N* = 32 without PTSD). Included participants were recruited and enrolled (between 11/2020 and 7/2024) at a single site, VA Portland Health Care System, with data contributed to the NAPS Consortium as well as banked into a local repository (VA MIRB #4086). iRBD, referring to those who were diagnosed with RBD before developing overt neurodegeneration, was diagnosed according to the International Classification of Sleep Disorders (2nd or 3rd Edition), including PSG. PTSD was determined via the PTSD checklist for the Diagnostic and Statistical Manual of Mental Disorders, Fifth Edition (PCL-5), following standard clinical criteria (i.e., total score >32) and subsequently confirmed through medical records or non-PTSD (total score ≤32)^19^. All study participants signed informed consent forms, and the study protocol was approved by the institutional review boards. This study was approved by the Faculty of Medicine Institutional Review Board A, Research Ethics Board Office at McGill University (approval number: 21-11-023) and was conducted in accordance with the Declaration of Helsinki. Clinical trial number: not applicable.

**Table 3:** Summary of cohorts and analytical methods used in the study

Study/Cohort	Number of cases	Number of controls	Data type	Analysis
Post-Traumatic Stress Disorder (PTSD) GWAS	137,136	1,085,746	GWAS summary statistics	MR, genetic correlation, PRS analysis
Idiopathic REM sleep behavior disorder (iRBD) GWAS	1061	8386	GWAS summary statistics	MR, genetic correlation
McGill cohort	1292	1353	GWAS Genotyping data	PRS analysis
NeuroGenetics Research Consortium (NGRC)	–	1968	GWAS Genotyping data	PRS analysis
National Institute of Neurological Disorders and Stroke (NINDS)	–	790	GWAS Genotyping data	PRS analysis
UK Biobank (UKB)	–	63,039	GWAS Genotyping data	PRS analysis

*GWAS* genome-wide association study, *MR* Mendelian randomization, *PRS* polygenic risk score.

### Genetic analysis

Genotyping of the McGill cohort was performed on the OmniExpress or Neurobooster arrays according to the manufacturer’s protocols (Illumina Inc). Pre-imputation quality control was completed as previously described (https://github.com/neurogenetics/GWAS-pipeline). Briefly, samples with heterozygosity outliers, call rate <95%, sex mismatch, non-European ancestry, or relatedness (pihat > 0.125) were excluded. SNPs were filtered for missingness ( < 0.05), disparate missingness between cases and controls (*p* > 1 × 10⁻⁴), missingness by haplotype (*p* > 1 × 10⁻⁴), and deviation from Hardy-Weinberg equilibrium in controls (*p* > 1 × 10⁻⁴). Imputation was performed using the TOPMed Imputation Server with the TOPMed reference panel r3 and default settings^20^. Imputed genotyping data for UKB controls were obtained from unrelated individuals of European ancestry (field 22006), randomly selected after excluding those diagnosed with mental and behavioral disorders (codes F00-F99) and nervous system diseases (codes G00-G99), based on International Classification of Diseases version-10 (ICD-10, field 41270). All samples were confirmed to be of European descent via principal component analysis (PCA), and post-imputation quality control was conducted using a standard protocol (https://choishingwan.github.io/PRS-Tutorial/target/), each cohort separately, to prepare the data for PRS analysis^21^.

We computed PTSD PRS for each iRBD case and each control using PRS continuous shrinkage (PRS-CS)^22^. Variants were selected via PRS-CS based on PTSD GWAS summary statistics, RBD genotyping data, and a European Linkage Disequilibrium reference panel recommended by PRS-CS (https://github.com/getian107/PRScs). Posterior SNP effect sizes were estimated using a continuous shrinkage approach within a Bayesian framework implemented by PRS-CS auto. Individual-level polygenic scores were computed using PLINK/2.0 (www.cog-genomics.org/plink/2.0)^23^ and Python 3.8.10 by averaging variant effect sizes within each chromosome and subsequently aggregating these values across chromosomes for each sample. We tested the association between PTSD polygenic scores and iRBD using a logistic regression model, adjusting for age, sex, cohort, and the first five principal components, using R version 4.1.2. As a sensitivity analysis, we derived a lifetime MDD PRS using publicly available polygenic scores (PGP000068)^24^ and included the MDD PRS as an additional covariate in the primary logistic regression model to evaluate whether the association between PTSD PRS and iRBD was independent of shared genetic liability with depression.

To estimate the genetic correlation between PTSD and iRBD, we employed LDSC^25^. Summary statistics for each dataset were preprocessed using the LDSC munge_sumstats.py script with an LD score reference panel derived from 1000 Genomes Phase 3 EUR ancestry (https://zenodo.org/records/10515792), ensuring the inclusion of high-quality SNPs. Subsequently, we utilized the ldsc.py script with the --rg flag to compute genetic correlations, applying the same LD score reference and the --no-intercept flag, as the PTSD and iRBD datasets had no sample overlap.

To investigate whether there is a causal link between PTSD and iRBD, we performed MR using TwoSampleMR package in R^26^. Genetic variants associated with PTSD (the exposure) that reached genome-wide significance (*p* ≤ 5e^-08^) were filtered from the *z*-score-based meta-analysis of the PTSD GWAS. Clumping was performed locally using PLINK/1.9 (www.cog-genomics.org/plink/1.9/)^23^ and an LD reference panel from 1000 Genomes Phase 3 EUR (https://zenodo.org/records/10515792) to select the SNP instruments. PTSD and iRBD data were then harmonized and MR was done using TwoSampleMR package to evaluate causality. The analysis was repeated with iRBD as the exposure and PTSD as the outcome to assess the causal impact of iRBD-associated genetics instruments on PTSD.

### Dat-SPECT analysis

Finally, thirty-eight consecutive iRBD participants with viable DaT-SPECT were included in retrospective analysis. DaT-SPECT abnormal status was determined via visual inspection by a blinded observer, a board-certified Neurologist with fellowship subspecialization in Movement Disorders (LEN). Chi-square analysis of the distribution of DaT-SPECT abnormal status was performed comparing PTSD and non-PTSD groups. Multiple logistic regression modeling was applied to control for potential confounders, specifically, (age, sex, history of traumatic brain injury, and time since RBD symptom onset).

## Data Availability

The iRBD summary statistics are available on GWAS catalog (https://www.ebi.ac.uk/gwas/, study accession GCST90204200). PTSD summary statistics are publicly available on PGC website (https://pgc.unc.edu/for-researchers/download-results/, accession ID ptsd2024). The UKB genotyping data were accessed through NeuroHub (https://www.mcgill.ca/hbhl/neurohub). Genotyping data from dbGaP (https://www.ncbi.nlm.nih.gov/gap/) are available through the NeuroGenetics Research Consortium (NGRC) (dbGaP accession: phs000196.v3.p1) and the National Institute of Neurological Disorders and Stroke (NINDS) Genome-Wide Genotyping in Parkinson’s Disease study (dbGaP accession: phs000089.v4.p2). Due to the sensitive nature of potentially identifiable protected health information of participants, deidentified clinical data will be made available upon request pursuant to institutional approvals for a Data Use Agreement or equivalent agreement, as appropriate.

## Code availability

The code supporting the findings of this study will be made publicly available on GitHub upon publication at: https://github.com/mghamg/PTSD-RBD-Study.
